# Phytohormone up-regulates the biochemical constituent, exopolysaccharide and nitrogen metabolism in paddy-field cyanobacteria exposed to chromium stress

**DOI:** 10.1186/s12866-020-01799-3

**Published:** 2020-07-13

**Authors:** Sanjesh Tiwari, Anuradha Patel, Sheo Mohan Prasad

**Affiliations:** Ranjan Plant physiology and Biochemistry Laboratory, Department of Botany, University of Allahabad, Prayagraj, 211002 India

**Keywords:** Chromium accumulation, Exopolysaccharides, Nitrogen metabolism, Phycobiliproteins, Scanning electron microscopy

## Abstract

**Background:**

Cyanobacteria are well known for their inherent ability to serve as atmospheric nitrogen fixers and as bio-fertilizers; however, increased contaminants in aquatic ecosystem significantly decline the growth and function of these microbes in paddy fields. Plant growth regulators play beneficial role in combating the negative effects induced by heavy metals in photoautotroph. Current study evaluates the potential role of indole acetic acid (IAA; 290 nm) and kinetin (KN; 10 nm) on growth, nitrogen metabolism and biochemical constituents of two paddy field cyanobacteria *Nostoc muscorum* ATCC 27893 and *Anabaena* sp*.* PCC 7120 exposed to two concentrations of chromium (Cr^VI^; 100 μM and 150 μM).

**Results:**

Both the tested doses of Cr^VI^ declined the growth, ratio of chlorophyll *a* to carotenoids (Chl *a*/Car), contents of phycobiliproteins; phycocyanin (PC), allophycocyanin (APC), and phycoerythrin (PE), protein and carbohydrate associated with decrease in the inorganic nitrogen (nitrate; NO_3_^—^ and nitrite; NO_2_^—^) uptake rate that results in the decrease in nitrate and ammonia assimilating enzymes; nitrate reductase (NR), nitrite reductase (NiR), glutamine synthetase (GS), glutamate synthase (GOGAT) except glutamate dehydrogenase (GDH). However, exogenous supplementation of IAA and KN exhibited alleviating effects on growth, nitrogen metabolism and exopolysaccharide (EPS) (first protective barrier against metal toxicity) contents in both the cyanobacteria, which probably occurred as a result of a substantial decrease in the Cr uptake that lowers the damaging effects.

**Conclusion:**

Overall result of the present study signifies affirmative role of the phytohormone in minimizing the toxic effects induced by chromium by stimulating the growth of cyanobacteria thereby enhancing its ability as bio-fertilizer that improved fertility and productivity of soil even in metal contaminated condition.

## Background

Industrial wastes have largely contributed to the deposition of toxic substances including heavy metals that contaminate the aquatic ecosystem. Among countless heavy metals, chromium (Cr) is predominantly present in the earth’s crust and exists in various oxidation states ranging from − 2 to + 6 [1] of which trivalent chromite (Cr^III^) and hexavalent chromate (Cr^VI^) are the most toxic and stable forms [2]. Upon comparing the degree of toxicity, it was found that Cr^VI^ due to its high solubility and mobility across biological membranes and as strong oxidizing agent, induces greater toxicity than Cr^III^ and also labelled as a potent carcinogen and mutagen that persists in soil for a longer time [3, 4]. The average concentration of Cr in the North Sea water is 0.7 μg L^− 1^ while in India the concentration is present below 2 μg L^− 1^ and in the drinking water it was up to 50 μg L^− 1^ [5]. However, the concentration has reach up to 5 g L^− 1^ due to release of Cr from various industries such as paint, metal finishing, steel manufacturing, chrome plating and leather tanning processes [2] that directly contaminate the river or canals.

Industrial effluents are directly disposed into rivers or canals that extensively used to irrigate the crop fields, by which toxic metals enter into the agricultural fields. Rice is a staple food crop that fulfills the food demand of over growing population, and rice fields provide a suitable environment for the growth of cyanobacteria during seedlings transplantation [6]. In paddy fields, cyanobacteria exclusively mediate the nitrogen fixation in form of ammonia (NH_4_^+^) thereby enhance the fertility of paddy fields and act as bio-fertilizer [7, 8]. Further, they are potent sources of carbohydrates, lipids, phenolics, vitamins, amino acids and sugars that directly or indirectly enhance the crop yield [9]. Chromium (Cr) induces negative effects on the growth of micro-flora associated with paddy fields, by obstructing their physiological and biochemical processes as studied by other workers in *Oscillatoria, Nostoc* and *Haematococcus* [10–12]. Excessive concentrations of Cr significantly decline the photosynthetic pigment contents [13], photosynthesis [14] and inorganic nitrogen uptake. Nitrogen (N) is a key macronutrient that regulates the growth and development of cyanobacteria as it is involved in the synthesis of nucleotides, amino acids, pigments, vitamins and enzymes [15, 16]. Chromium significantly declined the inorganic nitrogen uptake associated with decreased nitrogen and ammonia assimilating enzymes (NR, NiR, GS and GOGAT) except GDH (involved in alternative pathway of NH_4_^+^ assimilation) [17]. Earlier reports also indicated that biochemical constituents such as protein and carbohydrate were negatively affected by Cr stress [18, 19], beside this, EPS which is the polymer of carbohydrates and act primarily as a protective barrier against heavy metal stress were also found to decline under Cr stress.

Plants including micro-organisms survive in metal contaminated sites by secreting growth stimulating substances such as phytohormones that act as a signaling molecule and mediate growth under stress conditions [20]. Maintaining the micro-flora associated with paddy fields by the application of natural plant growth regulators is a innovative step towards understanding the role of plant hormones (auxins, gibberellins, cytokinins, abscisic acid, ethylene, and brassinosteroids) in cyanobacteria, by targeting an array of morphological, physiological and developmental processes. In plants, growth is mediated by auxin [21] while cytokinins regulate the process of cell division, and chloroplast development [22]. Similarly, in cyanobacteria several reports have been published concerning the presence and action of these phytohormones [23, 24]. Contrary to plants, the role of phytohormones in micro-algae is not yet clear. Thus, the present study is an attempt to explore a possible mechanism to interpret the ameliorating effects of phytohormones on physiological and biochemical attributes of *Nostoc muscorum* ATCC 27893 and *Anabaena* sp. PCC 7120 under chromium (Cr^VI^) stress which mainly prevails in real- crop field condition.

## Results

### Growth

**Fig. 1:**
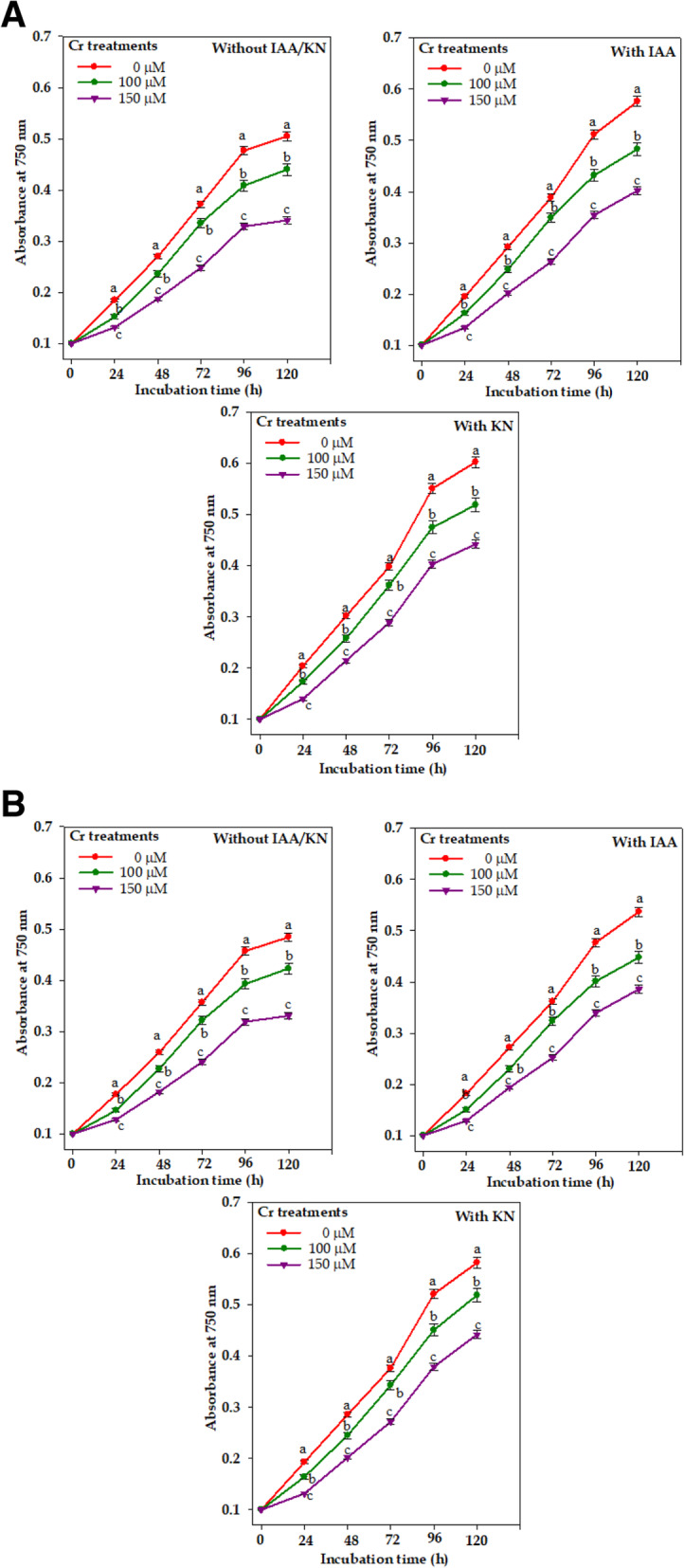
Dose response curve of *Nostoc muscorum* (**A**) and *Anabaena* sp. (**B**) exposed to different concentrations of chromium (Cr^VI^) with and without exogenous supplementation of phytohormones (Indole acetic acid; IAA and Kinetin; KN). Data are means±standard error of three replicates (*n* = 3). Lines followed by different letters how significant difference at *P* < 0.05 according to Duncan multiple range test (DMRT)

Result pertaining to growth (measured in terms of culture absorbance at 750 nm) of tested cyanobacteria i.e. *Nostoc muscorum* ATCC 27893 and *Anabaena* sp. PCC 7120 with and without exogenous supplemented IAA/KN has been presented in Fig. 1A and B. Chromium (Cr^VI^) under 100 and 150 μM declined the growth by 10 and 30% in *Nostoc muscorum* and 15 and 35% in *Anabaena* sp. that correspond to EC_10_ and EC_30_ for *N. muscorum* and EC_15_ and EC_35_ for *Anabaena* sp. as shown in growth response curve. Exogenous application of IAA along with Cr^VI^ (100 and 150 μM) resulted in reduction of growth by only 4 and 19% in *N. muscorum* and by 7 and 26% in *Anabaena* sp., respectively. Under similar condition, exogenous KN supplementation caused an inhibition of 1 and 14% in *N. muscorum* and 4 and 21% in *Anabaena* sp., respectively. The ameliorating effect of IAA and KN against Cr toxicity was greater in *N. muscorum* as compared to *Anabaena* sp.

### Chromium accumulation

**Fig. 2:**
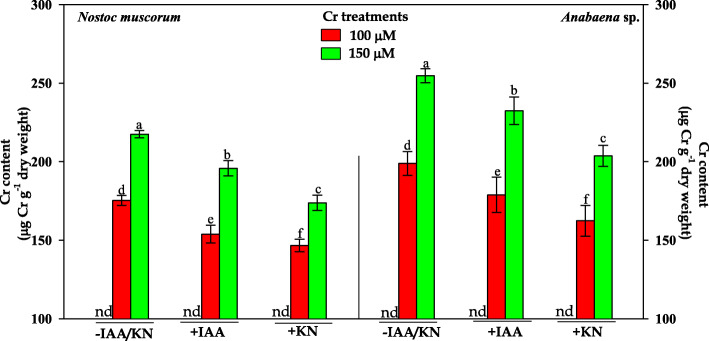
Impact of exogenous supplementation of phytohormones (Indole acetic acid; IAA and Kinetin; KN) on intracellular chromium accumulation of *Nostoc muscorum* and *Anabaena* sp. exposed to Cr^VI^ stress for 96 h. Data are means±standard error of three replicates (n = 3). Bars followed by different letters show significant difference at P < 0.05 according to Duncan multiple range test (DMRT). nd = not detected

The result related to the intracellular accumulation of Cr has been depicted in Fig. 2. The cellular accumulation of Cr was increased from 173 ± 3.0 μg Cr g^− 1^ dry weight to 216 ± 3.6 μg Cr g^− 1^ dry weight in *N. muscorum* and from 198 ± 2.6 μg Cr g^− 1^ dry weight to 267 ± 6.9 μg Cr g^− 1^ dry weight in *Anabaena* sp. when concentration of Cr was raised from 100 μM to 150 μM, respectively. Upon IAA/KN supplementation, the intracellular accumulation of Cr was significantly declined than the values recorded under-tested doses of Cr^VI^ (without the addition of phytohormones) and lowering in the cellular accumulation of Cr was more pronounced under KN supplementation.

### Photosynthetic pigments

Results pertaining to the ratio of chlorophyll *a* (Chl *a*) to carotenoids (Cars) and contents of phycobiliproteins in *N. muscorum* and *Anabaena* sp. under Cr^VI^ stress have been presented in Table 1. Inhibition in the Chl *a*/Cars ratio by 4 and 10% in *N. muscorum* and by 6 and 13% in *Anabaena* sp. was noticed under both the tested doses of Cr^VI^. Similarly, phycobiliproteins (PBPs; PC, APC and PE) were also found to be majorly affected under Cr^VI^ stress (Table 1). Among the three components of PBPs, the PC content was severely affected as it showed an inhibition of 14 and 37% in *N. muscorum* and 22 and 47% in *Anabaena* sp. at 100 and 150 μM of Cr^VI^, respectively. Under similar conditions, there is no significant reduction in the PE content while APC content followed a similar pattern. Further, exogenous supplementation of IAA/KN caused significant improvement in Chl *a*/Cars ratio by 2 and 4% in *N. muscorum* and 1 and 3% in *Anabaena* sp., *respectively*. However, with tested doses of Cr^VI^, improvement in the photosynthetic pigment contents were also recorded with the addition of phytohormones.

**Table 1:**
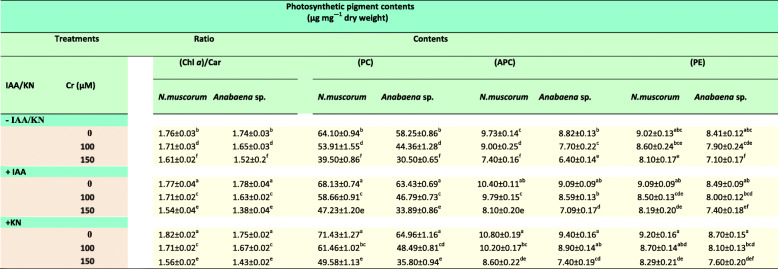
Impact of exogenous supplementation of phytohormones (Indole acetic acid; IAA and kinetin; KN) on photosynthetic pigment contents of *Nostoc muscorum* and *Anabaena* sp. exposed to Cr^VI^ stress

Data are means±standard error of three replicates (n = 3). Values followed by different superscript letters show significant difference according to Duncan multiple range test (DMRT) at P < 0.05 level

### Inorganic nitrogen uptake

For the estimation of inorganic nitrogen uptake both the cyanobacteria were grown in NO_3_^—^ and NO_2_^—^ containing BG-11 medium for 24 h prior to record NO_3_^—^ and NO_2_^—^ uptake rate and activity of nitrate reductase (NR) and nitrite reductase (NiR).

### Nitrate (NO_3_^—^) and nitrite (NO_2_^—^) uptake

**Fig. 3:**
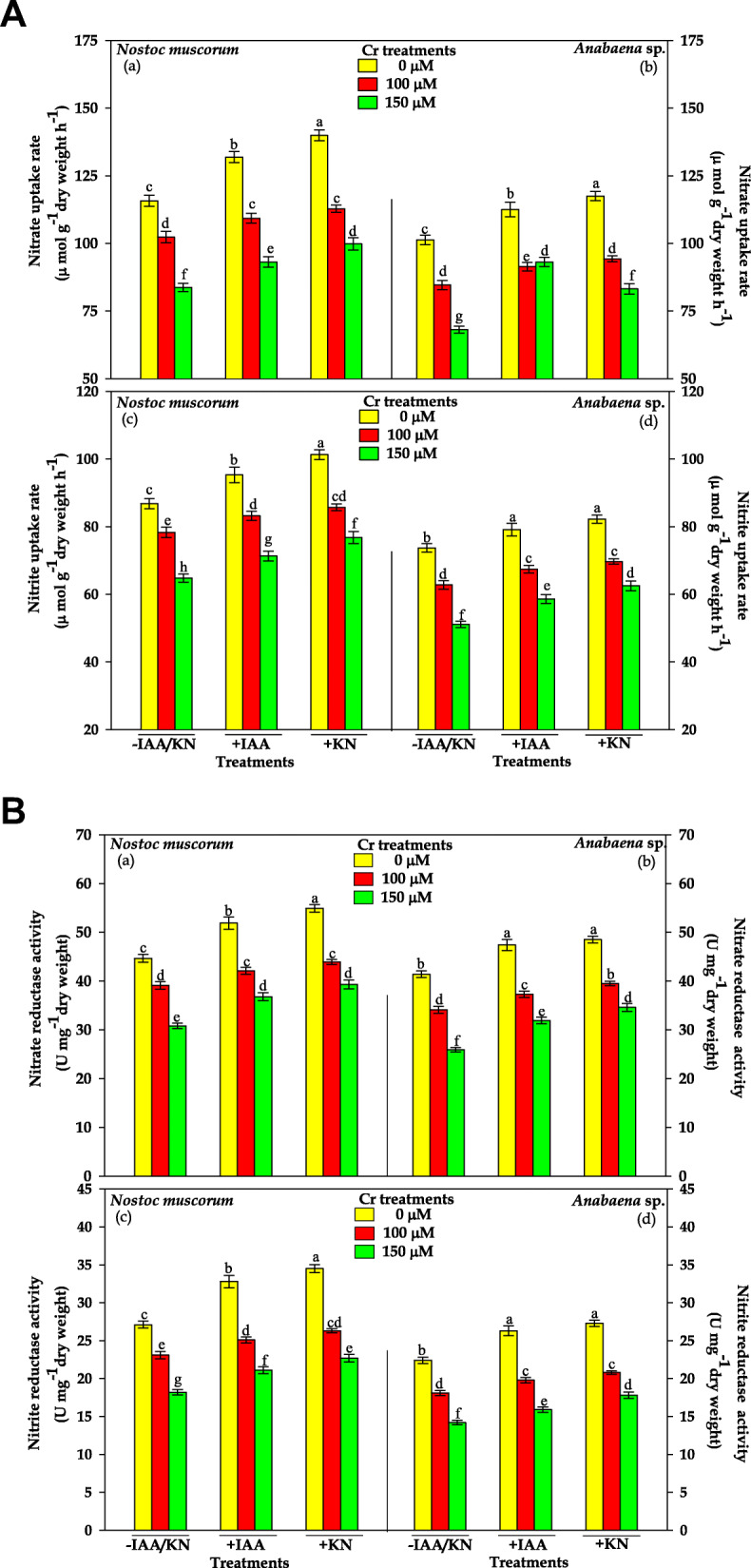
(**A**) Impact of exogenous supplementation of phytohormones (Indole acetic acid; IAA and Kinetin; KN) on nitrate and nitrite uptake rate of *Nostoc muscorum* and *Anabaena* sp. exposed to Cr^VI^ stress for 96 h. Data are means±standard error of three replicates (n = 3). Bars followed by different letters show significant difference at P < 0.05 according to Duncan multiple range test (DMRT). (**B**) Impact of exogenous supplementation of phytohormones (Indole acetic acid; IAA and Kinetin; KN) on nitrate and nitrite reductase activities of *Nostoc muscorum* and *Anabaena* sp. exposed to Cr^VI^ stress for 96 h. Data are means±standard error of three replicates (n = 3). Bars followed by different letters show significant difference at P < 0.05 according to Duncan multiple range test (DMRT)

Data related to the NO_3_^—^and NO_2_^—^uptake rate in both the tested cyanobacteria have been depicted in Fig. 3A. The results reveal that Cr^VI^ at 100 and 150 μM declined the uptake of NO_3_^—^ by 12 and 28% and NO_2_^—^ by10 and 25% in *N. muscorum*, and the corresponding decrease in NO_3_^—^ by 17 and 33% and NO_2_^—^ by 15 and 30% in *Anabaena* sp., respectively, over the control values. Under similar condition (100 and 150 μM Cr^VI^), exogenous supplementation of IAA exhibited alleviating effect as the reduction was only 6 and 20% for NO_3_^—^ uptake rate and 4 and 17% for NO_2_^—^uptake rate in *N. muscorum* while in *Anabaena* sp. it was 10 and 22% for NO_3_^—^ and 8 and 20% for NO_2_^—^uptake rate, respectively. Similar results were also obtained under KN treatments; however KN showed a greater alleviating effect as compared to IAA in both the cyanobacteria.

### Nitrate assimilating enzymes: nitrate and nitrite reductase activity

The NR and NiR activity in both the tested cyanobacteria has been shown in Fig. 3B. Results state that NR activity was inhibited by 13 and 31% in *N. muscorum* and by 18 and 38% in *Anabaena* sp. after 100 and 150 μM Cr^VI^ treatments, respectively. A similar pattern of inhibitory effect was noticed for NiR activity under Cr^VI^ stress (100 and 150 μM) showing 15 and 33% in *N. muscorum* and by 17 and 35% in *Anabaena* sp., respectively. Upon IAA supplementation, a significant recovery in the activity of both the enzymes was noticed, as it was decreased only by 6 and 18% in NR and by 7 and 22% in NiR in *N. muscorum*, and by10 and 23% in NR and 11 and 28% in NiR in *Anabaena* sp. at 100 and 150 μM Cr^VI^ treatments, respectively. Likewise, KN supplementation significantly alleviated the NR and NiR activity in both the cyanobacteria, however, the ameliorating effect was more pronounced in *N. muscorum* under Cr^VI^ stress.

### Ammonia assimilating enzymes

#### Glutamine synthetase and glutamate synthase (GS-GOGAT) activity

**Fig. 4:**
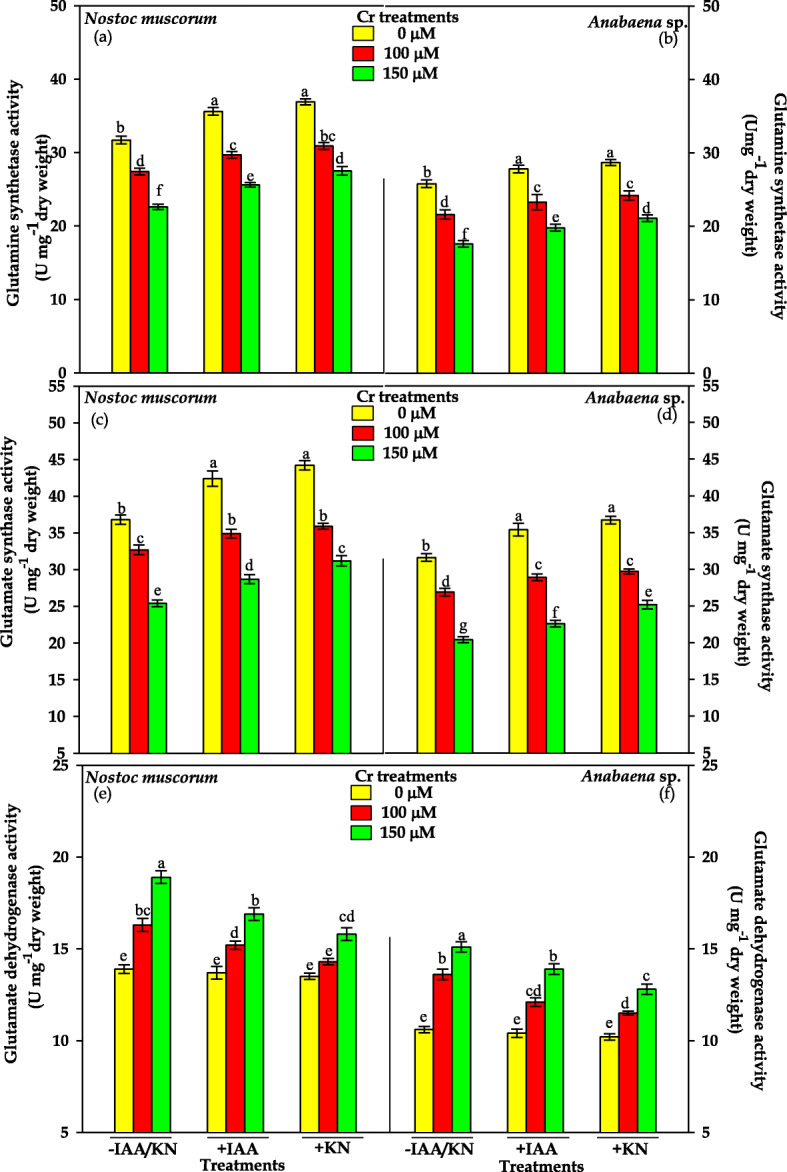
Impact of exogenous supplementation of phytohormones (Indole acetic acid; IAA and Kinetin; KN) on glutamine synthetase (**a**, **b**), glutamate synthase (**c**, **d**) and glutamate dehydrogenase (**e**, **f**) activities of *Nostoc muscorum* and *Anabaena* sp. exposed to Cr^VI^ stress for 96 h. Data are means±standard error of three replicates (n = 3). Bars followed by different letters show significant difference at P < 0.05 according to Duncan multiple range test (DMRT)

Results pertaining to the GS and GOGAT activity in Cr^VI^ stressed *N. muscorum* and *Anabaena* sp. supplemented with IAA and KN have been depicted in Fig. 4. Chromium at both the tested doses (100 and 150 μM) suppressed the activity of GS by 14 and 29% in *N. muscorum* and by 15 and 32% in *Anabaena* sp., respectively.

Likewise, similar doses of Cr^VI^ lowered down the GOGAT activity by 11 and 30% in *N. muscorum* and by 14 and 35% in *Anabaena* sp., respectively, over the values of the respective control. Exogenous supplementation of both the phytohormones IAA and KN caused significant improvement in the activity of both the enzymes and the alleviating effect against Cr^VI^ toxicity was greater in *N. muscorum.*

#### Glutamate dehydrogenase (GDH) activity

Data related to alternative GDH activity have been depicted in Fig. 4. As compared to the activity of other enzymes studied, a reverse trend was observed for GDH in Cr^VI^ stressed both cyanobacteria. Results exhibited that GDH activity was enhanced by 17 and 36% in *N. muscorum* and by 28 and 42% in *Anabaena* sp., respectively after 100 and 150 μM of Cr^VI^ treatments. Further, on IAA and KN supplementation to Cr^VI^ stressed cultures a declining trend in GDH activity was noticed in both the cyanobacteria, however, the activity was still considerably greater than that of control.

### Protein content

**Fig. 5:**
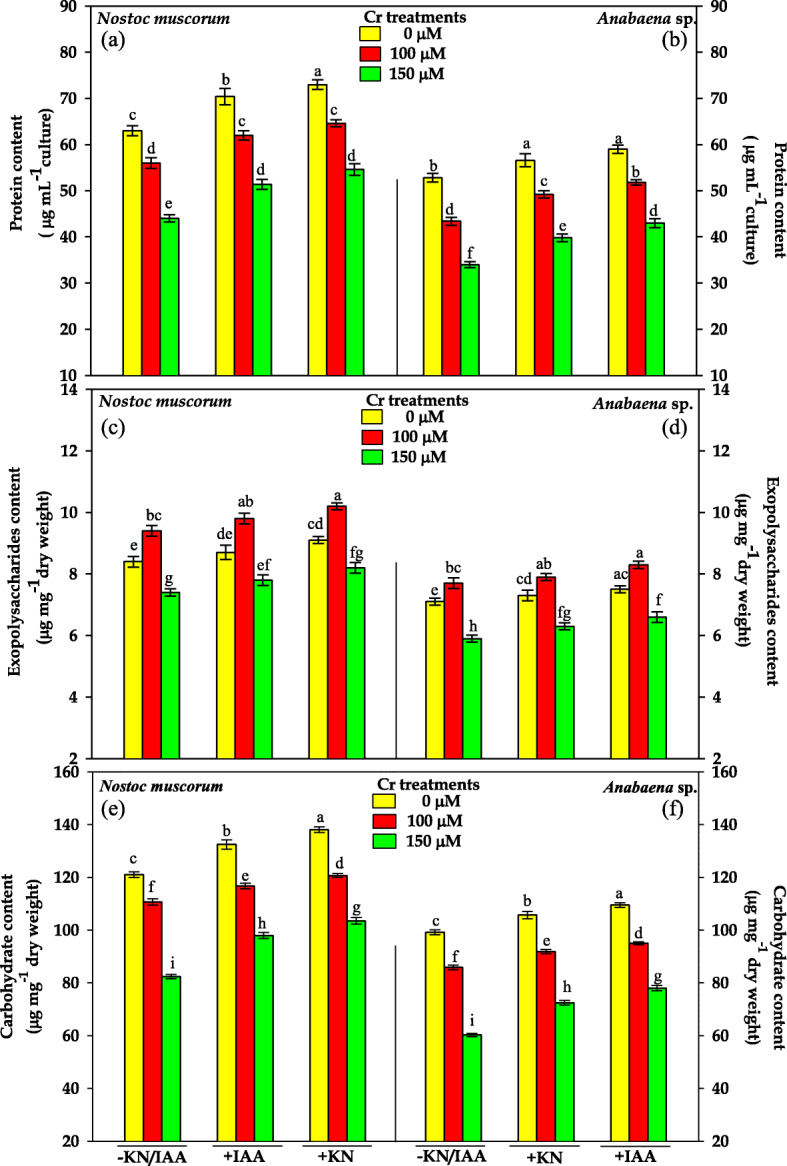
Impact of exogenous supplementation of phytohormones (Indole acetic acid; IAA and Kinetin; KN) on protein (**a**, **b**), exopolysaccharides (**c**, **d**) and carbohydrate contents (**e**, **f**) of *Nostoc muscorum* and *Anabaena* sp. exposed to Cr^VI^ stress for 96 h. Data are means±standard error of three replicates (n = 3). Bars followed by different letters show significant difference at P < 0.05 according to Duncan multiple range test (DMRT)

The results pertaining to the effect of IAA or KN supplementation on the protein content of Cr^VI^ stressed *N. muscorum* and *Anabaena* sp. have been portrayed in Fig. 5. The Cr^VI^ at 100 and 150 μM doses significantly decreased the protein content by 12 and 30% in *N. muscorum*, and by 18 and 35% in *Anabaena*, respectively, over the control values. However, IAA or KN supplementation to Cr^VI^ stressed cyanobacteria considerably lowered the inhibitory effect of Cr^VI^ on protein content but values were still less than control. Furthermore, the alleviating effect by the application of phytohormones was more pronounced in *N. muscorum*.

### Exopolysaccharides (EPS) content

The results pertaining to EPS content has been depicted in Fig. 5. Results reveal that at the lower dose of Cr^VI^ i.e. 100 μM, EPS content was enhanced by 11% in *N. muscorum*, and 8% in *Anabaena* sp., respectively. Contrary to this, 150 μM of Cr^VI^ caused a significant reduction in EPS content by 11% in *N. muscorum*, and 16% in *Anabaena* sp.*,* respectively, over the control values. Upon IAA or KN supplementation to Cr^VI^ stressed cyanobacterial cultures, EPS content was further enhanced at lower dose (100 μM Cr^VI^) and partial alleviation in EPS content was recorded at higher dose (150 μM Cr^VI^) in tested cyanobacteria.

### Carbohydrate content

The carbohydrate content in both the cyanobacteria was lowered at both the doses of Cr^VI^ (100 and 150 μM), as it was decreased by 13 and 19% in *N. muscorum*, and by 18 and 25% in *Anabaena* sp.*,* respectively (Fig. 5). The supplementation with the IAA or KN to Cr^VI^ treated cells, exhibited appreciable improvement in carbohydrate content in both the cyanobacteria but the values were still less than that of control.

### Scanning electron micrographs

**Fig. 6:**
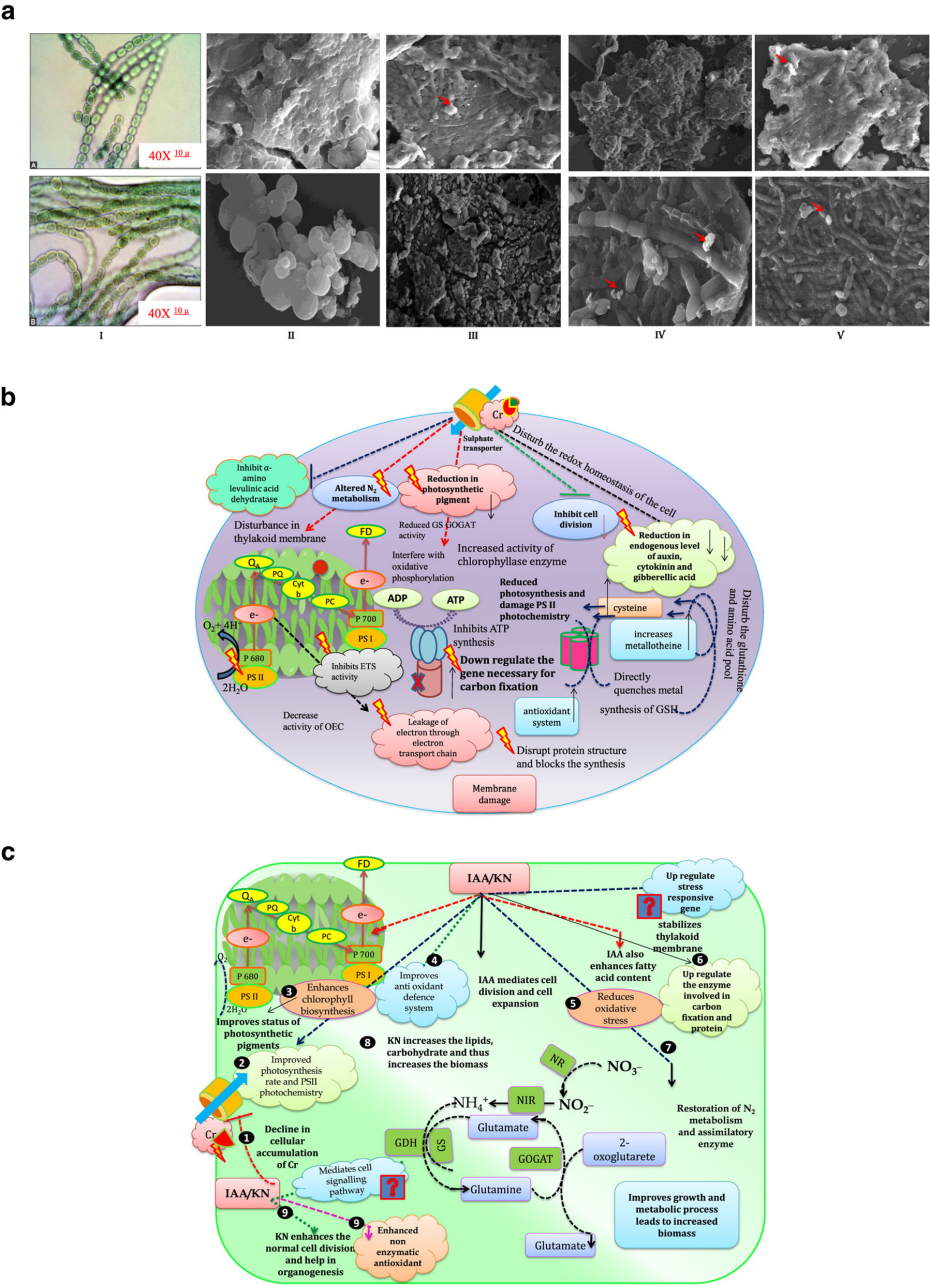
**a.** Scanning electron micrograph (SEM) of dry algal absorbent of (**A**) *Nostoc muscorum* and (**B**) *Anabaena* sp. treated with chromium with and without exogenous supplementation of (Indole acetic acid; IAA and Kinetin; KN); Cellular structure–I, without treatment (control) – II, chromium – III, IAA – IV, KN –V. **b.** Diagrammatic representation of toxicity mediated by chromium. **c** Up-regulation of antioxidant, nitrogen metabolism enzyme in alleviating Cr toxicity

SEM images clearly show presence of depression and groves on the surface of cyanobacteria that serve as binding sites for the metal ions. The white crusts over the apertures in the SEM images are evidently noticeable that supposed to be binding with the metal ions as indicated by red arrow. SEM images also show alteration in cell morphology as decrease in cell size and shrinkage was observed under Cr^VI^ stress. The white encrustations are more noticeable in *Anabaena* sp. as compared to *Nostoc muscorum* showing parallelism with the biochemical results (Fig. 6a).

## Discussion

Present study deals the positive effect of two novel phytohormones, auxin (indole-3-acetic acid; IAA) and cytokinin (6-furfuryl amino acid/kinetin, KN) on the growth, biochemical constituents and nitrogen metabolism (inorganic N uptake and assimilatory enzymes) of two paddy field cyanobacteria viz.*, Nostoc muscorum* ATCC 27893 and *Anabaena* sp. PCC 7120 subsequently exposed with hexavalent chromium (Cr^VI^; 100 and 150 μM). Chromium at both the tested doses exhibited a significant reduction in growth (Fig. 1a-b) in both cyanobacteria and this might be due to (i) increase in the intracellular Cr accumulation (Fig. 2) actively taken up *via* sulfate transporters [25], (ii) reduction in the photosynthetic pigment contents; Chl *a* and Car (published elsewhere) and phycobiliproteins (PBPs; PC, APC and PE) (Table 1), (iii) reduction in uptake rate of inorganic N and its assimilatory enzymes (nitrate and ammonia assimilation) except GDH (Figs. 3, 4), followed by the damaging effects on macromolecules such as protein, carbohydrate and exopolysaccharide (EPS) contents (Fig. 5). Our results are in agreement with earlier reports of Jayshree et al. [26] in *Oscillatoria,* Gupta et al. [27] in *Spirulina platensis* and Peng et al. [12] in *Haematococcus* where Cr significantly hampered the defense system and resulted in inhibition of growth as well as cell division [28]. Upon comparing the toxicity induced by Cr^VI^ in both cyanobacteria, *Anabaena* sp. showed greater toxicity as compared to *N. muscorum* that could be correlated with the more accumulation of Cr in the *Anabaena* cells (Fig. 2) which may be explained on the basis of absence of thick gelatinous sheath in case of *Anabaena* sp. [29]. Furthermore, adverse environmental conditions including metal toxicity decrease the endogenous level of phytohormones such as auxin, cytokinin and gibberellic acid which could also be correlated with growth retardation (Fig. 1) [30].

Negative effects induced by Cr^VI^ is minimized *via* exogenous supplementation of IAA/KN on all the studied parameters that could be correlated with (i) decrease in the intracellular Cr accumulation (Fig. 2), (ii) improvement in the light harvesting pigment contents; PBPs (Table 1), (iii) improvement in nitrate and ammonium assimilating enzymes (Figs. 3, 4) and biochemical constituents (Fig. 5). Under IAA supplementation, improvement in the growth under Cr^VI^ stress is not yet clearly known but it has been suggested that IAA positively participates in the cell division and cell expansion associated with improvement in the growth [31, 32]. IAA supplementation also enhances the fatty acid content in *Scendesmus* which might be another reason for growth improvement [33]. Similarly, KN also increased the lipid and carbohydrate in *Acutodesmus obliquus* as reported by Renuka et al. [34] associated with growth. In cyanobacteria *Nostoc entophytum, Hapalosiphon stuhlamanii* and *Nostoc muscorum* the KN induced improvement in growth and pigment contents were also reported by other workers [11, 35].

Upon IAA or KN supplementation the substantial decrease in intracellular Cr in tested cyanobacteria might have occurred due to the up-regulation of sulfate transporter protein which is primarily responsible for sulfate uptake inside the cell. Hence, under this condition there might have been greater competition between the uptake of sulfate and chromate resulting in an appreciable decline in intracellular Cr accumulation (Fig. 2).

Chromium at tested doses (100 and 150 μM) caused a substantial decrease in the ratio of Chl *a*/Cars and phycobiliproteins (PBPs) contents in cyanobacteria that play important role in photosynthesis [36]. Chromium declined the pigment contents either destroying its precursor or inhibiting the enzyme activity involved in pigment biosynthesis [37] or due to overproduction of ROS [19, 38]. Carotenoids are the accessory pigments and act as light-harvesting components and decrease in ratio of Chl *a*/Cars showed Cr^VI^ toxicity on photosynthetic pigments leading to damaging effect on functioning of the light-harvesting antenna complex. Furthermore, reduction in PBPs contents was noticed under Cr^VI^ stress (Table 1) and reduction is might be due to alteration at biosynthesis of PBPs or damage caused by Cr due to its easy accessibility for heavy metals as its location on exterior side of thylakoid membranes. Similar decrease in pigment content associated with growth retardation was also reported in *Spirulina* [39] *Synechocystis* [40] and in *Oscillatoria* under nitrogen starved condition [41]. Among all the three PBPs, PE was least affected under Cr^VI^ stress and the increasing order of damage followed the trend as- PE < APC < PC. Improvement in the pigment content under exogenous supplementation of IAA/KN to Cr^VI^ stressed cultures, could be attributed to (i) possible reduction in degradation of *δ*-aminolevulinic acid, (ii) stimulation in chlorophyll biosynthesis [42] and (iii) stabilization of thylakoid membrane [43]. Similar increase in ratio of Chl *a*/Cars and PBPs contents under IAA/KN supplementation was also noticed in earlier reports of Bajguz and Piotrowska-Niczyporuk [44] and Mansouri and Talebizadeh [45].

Being a macronutrient, nitrogen (N) is involved in the biosynthesis of various macromolecules (proteins and nucleic acids), thus the balance between uptake and assimilation of nitrogen is an important physiological process that has a direct impact on the growth of cyanobacteria. Photoautotroph (cyanobacteria) use nitrate (NO_3_^—^) as a source of nitrogen that are easily available to them and converted it into nitrite (NO_2_^—^) and ammonia (NH_4_^+^) *via* enzyme nitrate reductase (NR) and nitrite reductase (NiR). The inorganic N uptake rate and the activity of enzymes (NR and NiR) of both the tested cyanobacteria under Cr^VI^ stress was found to decrease, mainly due to reduced uptake of NO_3_^—^ (Fig. 3A). Active uptake of NO_3_^—^is mediated by ABC-type transporter which is ATP dependent [46] and reduction in uptake is might be due to declining ATP pool as Cr impairs the electron transport chain [47]. Similar results were also obtained by Sheeba et al. [48] in *Nostoc* and *Phormidium* and by Devriese et al. [49] in *Chlamydomonas* under metal, UV-B, pesticide stress. After entering into the cell, NO_3_^―^is reduced into NO_2_^―^ catalyzed by NR and then NO_2_^―^ is reduced into NH_4_^+^ by NiR, and Cr^VI^ significantly declined the activity of NR and NiR in a dose dependent manner due to increased cellular Cr accumulation (Fig. 2) but the effect was more pronounced in *Anabaena* sp. (Fig. 3 B). Our results are in agreement with Sangwan et al. [17] where Cr^VI^ significantly declined the nitrate assimilating enzymes. Further, reduction in NiR activity is might be due to (i) reduced carbon fixation, (ii) decreased NO_3_^―^ uptake, and (iii) altered electron transport that provides reduced Fd, an electron donor to reduce NO_2_^―^ [50].

After NO_3_^—^ assimilation, NH_4_^+^ is assimilated into amino acids *via* ammonia assimilating enzymes mainly by GS-GOGAT pathway [51]. In the current study, the GS/GOGAT activities were found to decrease significantly under Cr^VI^ stress (Fig. 4) that might be due to over-accumulation of free radicals that directly inhibited the enzyme activities [11]. For the activity of GS-GOGAT, ATP acts as cofactoe and Cr^VI^ causes impairment in ETC resulting in the low ATP synthesis and decreased enzyme activity was noticed [14]. Decrease in the activity of GS-GOGAT leads to the accumulation of NH_4_^+^ that causes growth reduction due to an altered intracellular pH which disturbs the osmotic balance and inhibited photosynthesis as reported in an earlier finding [52]. To overcome the ammonium toxicity, photoautotroph trigger the alternative ammonia assimilating enzyme glutamate dehydrogense (GDH) (Fig. 4) under stressful condition and also maintains the level of glutamate (involves in the synthesis of non-enzymatic antioxidants viz.*,* proline and phytochelatins). Supplementation of IAA/KN, appreciable increase in the uptake rate of NO_3_^—^ and NO_2_^—^ (Fig. 3 A), NO_3_^—^ and NH_4_^+^ assimilating enzymes in both the tested cyanobacteria (Fig. 3 B), that maintain the inorganic N content under Cr^VI^ stress (Fig. 1a-b). Improvement in GS-GOGAT activity under IAA/KN supplementation together with Cr^VI^ stress, lowered the GDH activity (high Km value) in comparison to Cr^VI^ treatment individually in both the tested cyanobacteria (Fig. 4) that could be explained on the basis of lesser availability of substrate i.e. NH_4_^+^, as it is mainly assimilated by GS/GOGAT pathway.

Cyanobacteria have immense potential to serve as valuable food products such as protein and carbohydrates that are directly associated with the growth under adverse environmental conditions. In the current study decline in protein content under Cr^VI^ stress (Fig. 5) might be due to the direct impact of Cr on protein synthesis, as observed by Shashirekha et al. [53] in Nostoc that supports the earlier findings where Cr induced inhibition of protein synthesis in *Oscillatoria* [40]. Furthermore, reduced level of carbohydrate was observed in the present study under-tested doses of Cr^VI^ in *N. muscorum* and *Anabaena* sp. (Fig. 5). Significant decrease in carbohydrate might have occurred due to reduction in the rate of photosynthesis and degradation of photosynthetic pigments [11], and our results are in parallel with the findings of Bajguz [54] in *Chlorella vulgaris* and in Gupta et al. [27] in *Spirulina platensis.*

Further, to overcome the negative effects induced by heavy metal, cyanobacteria secrete a high molecular weight polymer termed as exopolysaccharides (EPS) that acts as a physical barrier against heavy metals [55]. In the present study, a significant decrease in carbohydrate content might be associated with increase in EPS content at 100 μM Cr^VI^ (Fig. 5). Having positive charge on the Cr^VI^, the EPS could chelate the Cr^VI^ ion due to the presence of negative charge on its surface [56]. A similar increase in EPS content was also noticed in *Lyngbya* and *Nostoc linckia* and under Cr or Co stress [57, 58]. An increase in EPS content offers tolerance against heavy metal stress, but under excessive toxicity reduction in EPS content was noticed. Moreover, upon IAA/KN supplementation significant increase in EPS content was noticed (Fig. 5) associated with an increase in carbohydrate content. An increase in EPS contents suggests its role in removing Cr^VI^ from contaminated water and considered as “green “materials and behaves as bio-remediate, and our results of EPS secretion are confirmed by SEM images (Fig. 6a).

The two-way ANOVA test displays results to validate the interactive effect of Cr^VI^ and IAA/KN on growth in *N. muscorum* and *Anabaena* sp. The two-way ANOVA intricate that Cr^VI^ and IAA/KN alone significantly affected all the treatment, while in combination all the parameters showed insignificant relation (except NiR and GDH) hence, pointing towards relieving role of IAA/KN against Cr^VI^ induced toxicity in *N. muscorum* and *Anabaena* sp. (Table 1S).

## Conclusion

Findings suggest the noteworthy role of phytohormones; indole acetic acid (IAA) and kinetin (KN) in curtailing the negative effects induced by Cr^VI^ in paddy field cyanobacteria *N. muscorum* and *Anabaena* sp. Negative effects on growth, the ratio of Chl *a*/Cars and the contents of phycobiliproteins (PBPs) and nitrogen metabolism are considered easy target of heavy metal toxicity, and in the current study tested doses of Cr^VI^ caused damaging effects on these parameters. Further, Cr^VI^ also declined the biochemical constituents such as protein, carbohydrate and EPS contents. Exogenous supplementation of IAA/KN alleviated Cr^VI^ induced toxicity on growth and its related parameters such as inorganic N contents and improvement in nitrate and ammonia assimilating enzymes as well as protein content. Overall findings propose that phytohormones play major role in alleviating and enhancing the adaptation capability of tested cyanobacteria under Cr^VI^ stress and enhancing the N content in paddy fields that increases fertility and productivity of soil (Fig. 6b and c).

## Methods

### Growth conditions and treatment design

The cultures of *Nostoc muscorum* ATCC 27893 and *Anabaena* sp. PCC7120 were maintained in the laboratory under aseptic condition and unialgal cultures were obtained after serial dilution and the cells were examined frequently under light microscope. The cultures were grown in BG-11 medium at 25 ± 2 °C under 75 μmol photons m^− 2^ s^− 1^ in temperature controlled culture room. Further, screening experiment was conducted with chromium extending from 10 μM to 300 μM of Cr^VI^ (potassium dichromate; K_2_Cr_2_O_7_ as source) and on the basis of results two doses i.e. 100 and 150 μM of Cr^VI^ that inhibited the growth by 10 and 30% that correspond to EC10 and EC30 for *Nostoc muscorum*, and by 15 and 35% that correspond to EC15 and EC35 for *Anabaena* sp. were selected for the study. Similarly, single dose of IAA and KN were also screened out: IAA at 290 nM and KN at 10 nM enhanced the growth by 9 and 14% in *Nostoc muscorum* and by 7 and 10% in*Anabaena* sp.. Thus, considering the above screening following sets were prepared comprising of control (without Cr^VI^, KN and IAA supplementation), 100 μM Cr^VI^, 150 μM Cr^VI^, control+IAA, 100 μM Cr^VI^ + IAA and 150 μM Cr^VI^ + IAA, control+KN, 100 μM Cr^VI^ + KN and 150 μM Cr^VI^ + KN. There were three replicates (*n* = 3) for every treatment and all the parameters were analyzed after 96 h of experiment.

### Measurement of growth attributes

Growth was measured in terms of culture absorbance by taking absorbance at 750 nm by using UV-Visible Double beam-1700 Spectrophotometer.

### *Estimation of photosynthetic pigment contents* [59–61]

The amount of chlorophyll *a* and carotenoids was estimated by following the method of Porra et al. [59] and Goodwin [60], respectively by recording absorbance at 665 nm for chlorophyll *a* and at 450 nm for carotenoids with the help of UV-Visible Double beam-1700 Spectrophotometer, Shimadzu, Japan.

For the estimation of phycobiliproteins (PBPs), cells were treated with toluene and the water soluble pigments were extracted with 2.5 mM potassium phosphate buffer (pH 7.0) and the absorbance was read out at 615, 652 and 562nm and amount was quantified by using equation given by Bennett and Bogorad [61].

### Estimation of cellular accumulation of chromium

For intracellular accumulation of Cr, 80 ml of treated cyanobacterial cultures were centrifuged and pellets were washed with 1 mM EDTA and re-suspended in chilled phosphate buffer for 15 min to remove apo-plastic Cr and pellets were oven-dried at 70-80 °C for 3 days until it completely dries. Dried samples were digested by adding 5 ml of tri-acid mixture (HNO_3_, H_2_SO_4_ and HClO_4_ in ratio of 5:1:1) at 80 °C until a transparent solution obtained. The Cr was estimated by using atomic absorption spectrophotometer (iCE 3000 series, Model 3500 AAS, Thermo Scientific, UK). The instrument was calibrated by using standard solutions of Cr.

### *Estimation of NO*_*3*_^*—*^*and NO*_*2*_^*—*^*uptake* [62, 63]

For estimation of NO_3_^—^ and NO_2_^—^ uptake the cyanobacterial, cells were pre-incubated with 100 μM KNO_3_/ KNO_2_ under their respective growth conditions for 24 h and thereafter cells were harvested for uptake study.

#### Nitrate (NO_3_^—^) uptake

The NO_3_^—^ uptake rate in control and treated cyanobacterial cells were estimated by measuring the depletion of NO_3_^—^ from the external medium at 210 nm using the method of Cawse [62]. Samples were withdrawn after 4 h of incubation, subjected to centrifugation at 4000 *g* for 10 min and the cell-free supernatants were examined for residual NO_3_^—^.

#### Nitrite (NO_2_^—^) uptake

The NO_2_^—^ uptake rate was estimated by the depletion of NO_2_^—^ from the external medium through spectrophotometer at 540 nm using the method of Snell and Snell [63]. Samples were withdrawn after 4 h of incubation, subjected to centrifugation at 4000 *g* for 10 min and the cell-free supernatants were considered for residual NO_2_^—^.

### *Nitrate assimilating enzymes: estimation of nitrate reductase (NR) and nitrite reductase (NiR) activity* [64–66]

The NR/NiR activity was carried out with dithionite-reduced methyl viologen as a reductant in cells by adding mixed alkyltrimethyl ammonium bromide (MTA) to the reaction mixture according to the method of Herrero et al. [64–66]. The reaction mixture was incubated for 5 min at 25 °C, and NO_2_^—^ was estimated in corresponding cell-free media. For the measurement of NR and NiR activity in heterocystous cyanobacteria *N. muscorum* and *Anabaena* sp. were acclimatized in BG-11 medium containing KNO_3_ and NaNO_2_ at beginning of the experiment to induce NR and NiR enzymes, respectively. One unit of NR activity is defined as 1 nmol NO_2_^—^ formed min^− 1^ and one unit of NiR activity is defined as 1 nmol NO_2_^—^ consumed min^− 1^.

### *Ammonium assimilating enzymes activity* [67–70]

#### Estimation of glutamine synthetase (GS) activity

Glutamine synthetase (GS) activity was determined by following the method of Mérida et al. [67]. After centrifugation, cells were disrupted by sonication (Sonics Vibra Cell, Model VCX-130 PB, USA) and homogenate was centrifuged at 15000 *g* for 20 min at 4 °C (Model CPR-30, Remi, India) and the resulting supernatant constituted the cell extract. One unit of GS activity is defined as 1 nmol *γ*-glutamylhydroxamate formed min^− 1^.

#### Estimation of glutamate synthase (GOGAT) activity

The glutamine 2-oxoglutarate aminotransferase (GOGAT) activity was estimated by following the method of Meers et al. [68] and Navarro et al. [69]. After centrifugation, cells were re-suspended in Tris-HCl buffer (pH 7.6) and then disrupted thoroughly by sonication and centrifuged at 14000 *g* for 20 min at 4 °C and the supernatants were used as enzyme extract. Measuring the oxidation of NADH for *Nostoc muscorum* by taking absorbance at 340 nm and formation of glutamate for *Anabaena* sp. was used to determine the enzyme activity. One unit of GOGAT activity is defined as 1 nmol NADH oxidized min^− 1^ and 1 nmol glutamate formed min^− 1^.

#### Estimation of glutamate dehydrogenase (NADH-GDH) activity

Glutamate dehydrogenase activity (GDH) was quantified as per the method given by Chávez and Candau [70]. The cells were crushed in HEPES-NaOH buffer (pH 7.0) and the supernatant obtained was used as the enzyme extract. The reaction was started by the addition of NH_4_Cl and oxidation of NADH was recorded at 340 nm. One unit of GDH activity is defined as 1 nmol NADH oxidized min^− 1^.

### *Estimation of protein* [71]

The protein content of each sample was measured by the method of Bradford [71]. After centrifugation at 4000 g the pellets were homogenized with potassium phosphate buffer (pH 7.8) and centrifuged at 10,000 *g* for 10 min at 4 °C, and supernatants were used for the estimation of protein content by taking the absorbance at 595 NM.

### *Estimation of exopolysaccharides content* [72, 73]

Exopolysaccharides (EPS) content was determined by following the method of Sharma et al. [72]. The treated and untreated cultures were centrifuged at 3000 g for 15–20 min, and the cell-free suspension was taken and concentrated to 10 folds by evaporation at 40 °C. Followed by washing with isopropanol thrice and further left for drying at 37 °C the hydrolysates were analyzed for glucose by Dubois et al. [73] and the content was calculated as per the standard curve obtained for glucose.

### *Estimation of carbohydrate content* [73]

Carbohydrate content in each sample was estimated by adopting the method of Dubois et al. [73]. The samples were centrifuged at 10,000 *g* for 10 min. The sample was prepared of which 1.0 ml of suspension was added with 1.0 ml of 5% phenol and 5 ml H_2_SO_4_. The absorbance was recorded at 490 nm, using a spectrophotometer and compared with the standard curve prepared with pure glucose.

### Scanning electron micrography

Before and after Cr^VI^ exposition the surface morphology of the dry absorbent (exopolysaccharide) was studied by Scanning electron microscope (Double beam FEI Nova Nano SEM-450). After repeated washing in organic solvents, dry samples were mounted on stubs and coated with gold-palladium of thickness 100–1500 Å and then transferred to the sample chamber of the instrument. This was operated at 20 kV and 5.5 WD in high vacuum mode.

### Statistical analysis

Results were statistically analyzed, one-way ANOVA was performed to test significance level (Duncan multiple range tests, DMRT) at *p* < 0.05. Further, Two-way ANOVA test was also performed to show the differential action of Cr^VI^ and IAA/KN alone as well as in combination. The results presented are means ± standard error of three replicates (*n* = 3) and SPSS-16 software was used for DMRT.

## Supplementary information

**Additional file 1.**

## Data Availability

The data that support the findings of this study are available from but restrictions apply to the availability of these data, which were used under license for the current study, and so are not publicly available. Data are however available from the authors upon reasonable request and with permission of Prof. Sheo Mohan Prasad.

## Abbreviations

APC: Allophycocyanin
Cars: Carotenoids
Chl *a*: Chlorophyll a
Cr: Chromium
EPS: Exopolysachharides
GDH: Glutamate dehydrogenase
GOGAT: Glutamate synthase
GS: Glutamine synthetase
IAA: Indole acetic acid
KN: Kinetin
NH_4_^+^: Ammonia
NiR: Nitrite reductase
NO_2_^—^: Nitrite
NO_3_^—^: Nitrate
NR: Nitrate reductase
PBPs: Phycobiliprpteins
PC: Phycocyanin
PE: Phycoerythrin
ROS: Reactive oxygen species
SEM: Scanning electron microscope
