# Supplementary material for: Phytohormone up-regulates the biochemical constituent, exopolysaccharide and nitrogen metabolism in paddy-field cyanobacteria exposed to chromium stress
Source: BMC Microbiol. 2020 Jul 13;20:206. doi: 10.1186/s12866-020-01799-3 (PMC7359020; doi:10.1186/s12866-020-01799-3)
Supplement: Supplementary file 1 — Additional file 1. [file 12866_2020_1799_MOESM1_ESM.docx]

**Table 1S-A.** Two–way ANOVA results for the individual and interactive effect of Cr^VI^ and IAA on growth and growth regulating parameters of *Nostoc muscorum*.

| **Variables** | |  | **df** | **F** | **P value** |  | **df** | **F** | **P value** |  | **df** | **F** | **P value** |
| --- | --- | --- | --- | --- | --- | --- | --- | --- | --- | --- | --- | --- | --- |
|  | |  | **Growth** | | |  | **PC content** | | |  | **Nitrate uptake** | | |
| Cr | |  | 2 | 151.3 | **<0.000** |  | 2 | 102.1 | **<0.001** |  | 2 | 102.1 | **<0.001** |
| IAA | |  | 1 | 34.58 | **<0.001** |  | 1 | 25.03 | **<0.000** |  | 1 | 25.03 | **<0.000** |
| Cr*IAA | |  | 2 | 2.440 | **<0.128** |  | 2 | 0.588 | **0.571** |  | 2 | 0.588 | **0.571** |
|  |  | | | | | | | | | | | | |
|  | |  | **Nitrite uptake** | | |  | **NR activity** | | |  | **NiR activity** | | |
| Cr | |  | 2 | 130.7 | **<0.001** |  | 2 | 184.9 | **<0.001** |  | 2 | 133.0 | **<0.001** |
| IAA | |  | 1 | 36.10 | **<0.000** |  | 1 | 61.73 | **<0.000** |  | 1 | 54.72 | **<0.000** |
| Cr*IAA | |  | 2 | 2.048 | 0.172 |  | 2 | 7.332 | 0.008 |  | 2 | 3.165 | **0.079** |
|  |  | | | | | | | | | | | | |
|  | |  | **GS activity** | | |  | **GOGAT activity** | | |  | **GDH activity** | | |
| Cr | |  | 2 | 131.6 | **<0.001** |  | 2 | 166.8 | **<0.001** |  | 2 | 86.09 | **<0.001** |
| IAA | |  | 1 | 39.84 | **<0.001** |  | 1 | 43.43 | **<0.001** |  | 1 | 18.52 | **<0.001** |
| Cr*IAA | |  | 2 | 0.750 | 0.493 |  | 2 | 3.183 | 0.078 |  | 2 | 4.157 | **0.042** |
|  | | | | | | | | | | | | | |
|  | |  | **Protein** | | |  | **exopolysaccharide** | | |  |  |  |  |
| Cr | |  | 2 | 178.5 | **<0.001** |  | 2 | 78.70 | **<0.001** |  |  |  |  |
| IAA | |  | 1 | 44.49 | **<0.001** |  | 1 | 5.647 | **<0.035** |  |  |  |  |
| Cr* IAA | |  | 2 | 0.750 | 0.493 |  | 2 | 0.353 | 0.710 |  |  |  |  |
|  | | | | | | | | | | | | | |

The bold P values show significant level.

**Table 1S-B.** Two–way ANOVA results for the individual and interactive effect of Cr^VI^ and KN on growth and growth regulating parameters of *Nostoc muscorum*.

| **Variables** | |  | **df** | **F** | **P value** |  | **df** | **F** | **P value** |  | **df** | **F** | **P value** |
| --- | --- | --- | --- | --- | --- | --- | --- | --- | --- | --- | --- | --- | --- |
|  | |  | **Growth** | | |  | **PC content** | | |  | **Nitrate uptake** | | |
| Cr | |  | 2 | 124.3 | **<0.001** |  | 2 | 226.4 | **<0.001** |  | 2 | 131.3 | **<0.001** |
| KN | |  | 1 | 62.50 | **<0.001** |  | 1 | 39.47 | **<0.000** |  | 1 | 92.10 | **<0.001** |
| Cr*KN | |  | 2 | 2.690 | **<0.108** |  | 2 | 1.677 | 0.459 |  | 2 | 3.060 | 0.084 |
|  |  | | | | | | | | | | | | |
|  | |  | **Nitrite uptake** | | |  | **NR activity** | | |  | **NiR activity** | | |
| Cr | |  | 2 | 178.0 | **<0.001** |  | 2 | 209.6 | **<0.001** |  | 2 | 156.8 | **<0.001** |
| KN | |  | 1 | 117.2 | **<0.001** |  | 1 | 145.0 | **<0.000** |  | 1 | 125.8 | **<0.000** |
| Cr*KN | |  | 2 | 6.466 | 0.012 |  | 2 | 9.100 | 0.004 |  | 2 | 5.329 | **0.022** |
|  |  | | | | | | | | | | | | |
|  | |  | **GS activity** | | |  | **GOGAT activity** | | |  | **GDH activity** | | |
| Cr | |  | 2 | 162.3 | **<0.001** |  | 2 | 216.0 | **<0.000** |  | 2 | 84.93 | **<0.001** |
| KN | |  | 1 | 118.0 | **<0.001** |  | 1 | 130.1 | **<0.001** |  | 1 | 63.83 | **<0.001** |
| Cr*KN | |  | 2 | 1.390 | 0.285 |  | 2 | 6.523 | 0.012 |  | 2 | 11.713 | **0.002** |
|  | | | | | | | | | | | | | |
|  | |  | **Protein** | | |  | **exopolysaccharide** | | |  |  |  |  |
| Cr | |  | 2 | 164.4 | **<0.001** |  | 2 | 92.38 | **<0.001** |  |  |  |  |
| IAA | |  | 1 | 132.2 | **<0.001** |  | 1 | 40.69 | **<0.001** |  |  |  |  |
| Cr* KN | |  | 2 | 0.490 | 0.624 |  | 2 | 0.077 | 0.926 |  |  |  |  |
|  | | | | | | | | | | | | | |

The bold P values show significant level.

**Table 1S-C.** Two–way ANOVA results for the individual and interactive effect of Cr^VI^ and KN on growth and growth regulating parameters of *Anabaena* sp.

| **Variables** | |  | **df** | **F** | **P value** |  | **df** | **F** | **P value** |  | **df** | **F** | **P value** |
| --- | --- | --- | --- | --- | --- | --- | --- | --- | --- | --- | --- | --- | --- |
|  | |  | **Growth** | | |  | **PC content** | | |  | **Nitrate uptake** | | |
| Cr | |  | 2 | 196.8 | **<0.001** |  | 2 | 447.0 | **<0.001** |  | 2 | 162.5 | **<0.001** |
| IAA | |  | 1 | 59.07 | **<0.001** |  | 1 | 48.02 | **<0.000** |  | 1 | 110.2 | **<0.001** |
| Cr*IAA | |  | 2 | 4.252 | **<0.001** |  | 2 | 0.760 | 0.489 |  | 2 | 1.884 | 0194 |
|  |  | | | | | | | | | | | | |
|  | |  | **Nitrite uptake** | | |  | **NR activity** | | |  | **NiR activity** | | |
| Cr | |  | 2 | 226.5 | **<0.001** |  | 2 | 0.256 | **<0.001** |  | 2 | 184.6 | **<0.001** |
| IAA | |  | 1 | 86.67 | **<0.001** |  | 1 | 133.4 | **<0.000** |  | 1 | 131.3 | **<0.001** |
| Cr*IAA | |  | 2 | 2.381 | 0135 |  | 2 | 3.421 | 0.087 |  | 2 | 2.015 | 0.176 |
|  |  | | | | | | | | | | | | |
|  | |  | **GS activity** | | |  | **GOGAT activity** | | |  | **GDH activity** | | |
| Cr | |  | 2 | 191.4 | **<0.001** |  | 2 | 270.9 | **<0.000** |  | 2 | 118.6 | **<0.001** |
| IAA | |  | 1 | 83.69 | **<0.001** |  | 1 | 113.0 | **<0.001** |  | 1 | 71.25 | **<0.001** |
| Cr*IAA | |  | 2 | 0.707 | 0.513 |  | 2 | 3.287 | 0.073 |  | 2 | 10.11 | **0.003** |
|  | | | | | | | | | | | | | |
|  | |  | **Protein** | | |  | **exopolysaccharide** | | |  |  |  |  |
| Cr | |  | 2 | 224.2 | **<0.001** |  | 2 | 82.14 | **<0.001** |  |  |  |  |
| IAA | |  | 1 | 137.4 | **<0.001** |  | 1 | 25.50 | **<0.001** |  |  |  |  |
| Cr* IAA | |  | 2 | 1.609 | 0.240 |  | 2 | 0.618 | 0.5 |  |  |  |  |
|  | | | | | | | | | | | | | |

The bold P values show significant level.

**Table 1S-D.** Two–way ANOVA results for the individual and interactive effect of Cr^VI^ and IAA on growth and growth regulating parameters of *Anabaena* sp.

| **Variables** | |  | **df** | **F** | **P value** |  | **df** | **F** | **P value** |  | **df** | **F** | **P value** |
| --- | --- | --- | --- | --- | --- | --- | --- | --- | --- | --- | --- | --- | --- |
|  | |  | **Growth** | | |  | **PC content** | | |  | **Nitrate uptake** | | |
| Cr | |  | 2 | 256.5 | **<0.000** |  | 2 | 570.3 | **<0.001** |  | 2 | 130.8 | **<0.001** |
| IAA | |  | 1 | 29.93 | **<0.001** |  | 1 | 28.46 | **<0.000** |  | 1 | 28.73 | **<0.000** |
| Cr*IAA | |  | 2 | 3.219 | <0.076 |  | 2 | 1.048 | 0.381 |  | 2 | 0.632 | **0.549** |
|  |  | | | | | | | | | | | | |
|  | |  | **Nitrite uptake** | | |  | **NR activity** | | |  | **NiR activity** | | |
| Cr | |  | 2 | 162.7 | **<0.001** |  | 2 | 249.7 | **<0.001** |  | 2 | 178.7 | **<0.001** |
| IAA | |  | 1 | 43.35 | **<0.000** |  | 1 | 52.99 | **<0.000** |  | 1 | 55.84 | **<0.000** |
| Cr*IAA | |  | 2 | 1.053 | 0.379 |  | 2 | 3.648 | 0.058 |  | 2 | 1.198 | 0.335 |
|  |  | | | | | | | | | | | | |
|  | |  | **GS activity** | | |  | **GOGAT activity** | | |  | **GDH activity** | | |
| Cr | |  | 2 | 156.4 | **<0.001** |  | 2 | 230.1 | **<0.001** |  | 2 | 125.4 | **<0.001** |
| IAA | |  | 1 | 24.43 | **<0.001** |  | 1 | 34.04 | **<0.001** |  | 1 | 21.75 | **<0.001** |
| Cr*IAA | |  | 2 | 0.159 | 0.855 |  | 2 | 1.553 | 0.251 |  | 2 | 3.595 | **0.060** |
|  | | | | | | | | | | | | | |
|  | |  | **Protein** | | |  | **exopolysaccharide** | | |  |  |  |  |
| Cr | |  | 2 | 178.5 | **<0.001** |  | 2 | 78.70 | **<0.001** |  |  |  |  |
| IAA | |  | 1 | 44.49 | **<0.001** |  | 1 | 5.647 | **<0.035** |  |  |  |  |
| Cr* IAA | |  | 2 | 0.750 | 0.493 |  | 2 | 0.353 | 0.710 |  |  |  |  |
|  | | | | | | | | | | | | | |

The bold P values show significant level.
